# Mitigating the Risk of Transfusion-Transmitted Dengue in Australia

**DOI:** 10.1155/2016/3059848

**Published:** 2016-11-13

**Authors:** Kelly Rooks, Clive R. Seed, Jesse J. Fryk, Catherine A. Hyland, Robert J. Harley, Jerry A. Holmberg, Denese C. Marks, Robert L. P. Flower, Helen M. Faddy

**Affiliations:** ^1^Research and Development, Australian Red Cross Blood Service, Brisbane, QLD, Australia; ^2^Medical Services, Australian Red Cross Blood Service, Perth, WA, Australia; ^3^Medical Services, Australian Red Cross Blood Service, Brisbane, QLD, Australia; ^4^Grifols Diagnostic Solutions, Inc., Emeryville, CA, USA; ^5^Research and Development, Australian Red Cross Blood Service, Sydney, NSW, Australia; ^6^School of Medicine, The University of Queensland, Brisbane, QLD, Australia

## Abstract

Dengue viruses (DENV 1–4) are a risk to transfusion safety, with several transfusion-transmitted (TT) cases reported globally. DENV 1–4 are endemic in over 100 countries, with seasonal outbreaks occurring in northeastern Australia. To mitigate TT-DENV risk in Australia, fresh blood components are not manufactured from donors returning from any area (domestic/overseas) with known dengue transmission. Alternatively, TT-DENV risk may be mitigated using an appropriate blood donor screening assay. We aimed to determine the rate of dengue infection in donors during dengue outbreaks in Australia. Plasma samples were collected from blood donors during local dengue outbreaks. All samples were tested for the presence of DENV RNA and selected samples were tested for DENV antigen (nonstructural protein 1, NS1) with two assays. No donors residing in high risk areas had detectable levels of DENV RNA or NS1 and no cases of DENV viremia were detected in blood donors residing in areas of Australia experiencing DENV outbreaks. Definitive conclusions could not be drawn from this study; however, the lack of detection of DENV RNA or antigen in donations suggests that the current risk of TT-DENV is low and maintaining the fresh component restriction for “at-risk” donors is appropriate.

## 1. Introduction

Dengue is one of the most important arboviral pathogens worldwide, with an estimated 390 million infections per year [1]. Of these estimated dengue infections, only 96 million manifest clinically, with the majority of infections therefore asymptomatic [2]. Dengue is emerging or reemerging across the globe, with transmission occurring in over 100 countries each year [3].

There are four serotypes of dengue virus (DENV): DENV-1, DENV-2, DENV-3 and DENV-4. DENV are mosquito-borne, with the primary vector being* Aedes aegypti*. This urban-adapted mosquito is distributed throughout tropical and subtropical climates, giving rise to endemic and epidemic DENV transmission in both developing and developed nations [4]. A secondary vector capable of transmitting DENV,* Aedes albopictus*, has increased in geographic range in recent years, which may contribute to the increasing number of dengue infections [4].

Almost 75% of the DENV global disease burden is in the Southeast Asian and Western Pacific Regions [5]. In Australia, seasonal outbreaks occur in the northeast of the country [1, 6]. One of the largest DENV epidemics in Australia's history occurred in 2008/2009, with distinct outbreaks in Cairns, Innisfail, and Townsville [7]. Collectively, this epidemic resulted in over 1,000 confirmed infections. Another sizeable DENV outbreak occurred in northeastern Australia in the summer of 2012/2013, resulting in 534 confirmed cases [8].

Given the high rate of asymptomatic DENV infection, this virus poses a risk to transfusion safety [9]. Transfusion transmitted-DENV (TT-DENV) has been reported in Singapore, Hong Kong, Puerto Rico, and Brazil [10–13]. To date, no cases of TT-DENV have occurred in Australia. The incidence of TT-DENV is likely to be higher than what has been published, due to underreporting. Moreover, DENV viremia has been detected in blood donors from Honduras, Puerto Rico, and Brazil, reinforcing the potential risk of TT-DENV [12, 14, 15].

To help mitigate the risk of TT-DENV in Australia, donors are unable to donate fresh blood components for 4 weeks upon their return from countries endemic for DENV or areas in northern Australia experiencing dengue outbreaks [16]. Plasma may still be collected during this 4-week restrictive period if destined for fractionation, as the manufacturing process includes viral inactivation steps that have been shown to effectively inactivate DENV, allowing plasma derivatives to be considered safe with respect to this virus [17]. Currently, there is no approved DENV test in Australia for blood screening, and although some pathogen inactivation (PI) technologies have been demonstrated to effectively inactivate DENV in plasma and platelet components [18–21] these methods are not approved for use in Australia at present. Our approach of restricting donations from “at-risk” individuals results in fresh component losses and considerable cost, which may potentially impact on the ability to meet clinical demand [22]. However, this approach is deemed suitable in the absence of other approved risk mitigation strategies.

It is clear that DENV poses a risk to the safety of Australia's blood supply, which may justify these relatively high-cost risk-reduction strategies. However, alternative testing technologies for DENV detection may be utilised for donor screening, if deemed appropriate and licenced for such use. Therefore, this study aimed to determine the rate of viremia in Australian blood donors during local dengue outbreaks, by testing plasma samples for the presence of DENV RNA and DENV antigen (nonstructural protein 1, NS1).

## 2. Materials and Methods

### 2.1. Sample Collection

Samples were collected from donors in North Queensland during two dengue outbreaks: 2008/2009 (*n* = 973) and 2012/2013 (*n* = 5,518). For samples collected during the 2008/2009 outbreak, an extra sample was collected from all donations during the outbreak. These samples were collected in plasma preparation tubes (PPT, BD Vacutainer Plasma Preparation Tubes, Becton Dickinson, Plymouth, UK) and centrifuged at a relative centrifugal force (RCF) of 1,100 for 10 minutes as per routine procedure. Demographic data were obtained for all donations to allow identification of donors at “higher risk” of exposure to DENV, defined as residence in areas of Cairns that reported more than 20 laboratory confirmed dengue cases. Samples from 2012/2013 were collected from both Cairns and Townsville during the dengue outbreak. Additional control samples (*n* = 1,601) were obtained from Melbourne in southern Australia in 2012/2013, where transmission of DENV does not occur. Samples collected in 2012/2013 were recovered after routine testing was completed, representing a convenience sample. Samples from 2012/2013 were collected into ethylenediaminetetraacetic acid (EDTA) spray-coated tubes (BD Vacutainer Whole Blood Collection Tube with Spray-Coated K_2_EDTA, Becton Dickinson) and centrifuged at 1,258 RCF for 10 minutes as per routine procedure. All samples were stored at −20°C until testing. This study was carried out under approval by the Blood Service Human Research Ethics Committee.

### 2.2. Dengue NS1 Testing

Samples from donors residing in “higher-risk” areas during the 2008/2009 DENV outbreak (*n* = 973) were tested for the presence of DENV NS1 using both the PanBio Dengue Early ELISA (Alere, Brisbane, Queensland, Australia) and the Platelia Dengue NS1 Ag Kit (Bio-Rad, Hercules, CA, USA) as per the manufacturer's instructions, which included positive, negative, and internal controls. Both kits utilised a one-step sandwich format ELISA for the detection of DENV NS1 in either plasma or serum. Samples were first tested in singlicate on both assays, with initial reactive or equivocal samples being retested in duplicate. Samples were only classified positive if they were reactive 2 or 3 times on both assays. Results from the PanBio Dengue Early ELISA Kit were calculated in “PanBio units” and considered negative if the results were <9, equivocal if 9–11, and positive if ≥11 (sensitivity: 72.3% and specificity: 100% [23]). The Platelia Dengue NS1 Ag Kit results were calculated in ratios and considered negative if results were <0.5, equivocal if between 0.5 and 1, and positive if ≥1 (sensitivity: 83.6% and specificity: 98.7% [23]).

### 2.3. Dengue RNA Testing

The following samples were tested for the presence of DENV RNA: 664 samples from higher-risk areas during the 2008/2009 DENV outbreak (representing all samples remaining with an adequate volume); 5,518 samples from the 2012/2013 DENV outbreak; and 1,601 control samples from southern Australia. Samples were tested with a Procleix DENV assay on a Procleix Panther System (Grifols Diagnostic Solutions, Inc., Emeryville, CA, USA, and Hologic, San Diego, CA, USA) as per manufacturer's instructions, which included positive, negative, and internal controls, at the American Red Cross laboratories in Charlotte, North Carolina. The Procleix DENV assay is based on transcription mediated amplification (TMA) and can detect all four DENV serotypes [4]. The 95% limit of detection is reported to be approximately 15 copies/mL (95% CI, 11.5–20.9 copies/mL), with a specificity of >99.91% [4].

### 2.4. Analyses

Data were stored using Microsoft Excel 2010 (Microsoft, Redmond, WA, USA) databases and analyses were also performed using this software. Individual proportions were calculated, along with the corresponding exact 95% confidence intervals (CI) using a standard method [24]. Specifically, for zero-risk estimates, the 95% CIs were calculated as follows:

 Upper 95% CI = 1 − 0.025^(1/*n*)^, where *n* = number of samples tested.
 Lower 95% CI = 0.

## 3. Results

Samples from areas of Cairns with higher numbers of confirmed DENV cases during the 2008/2009 outbreak were selected for DENV NS1 antigen testing (*n* = 973). Of the samples tested, 32 were positive (overall either 2/3 or 3/3) with the PanBio Dengue Early ELISA (Table 1). Using the Platelia Dengue NS1 Ag Kit, only one sample tested initially equivocal; however, it was negative on duplicate repeat testing (Table 1). As no samples tested positive on both assays, all samples were deemed negative for DENV antigen.

Samples collected from the 2008/2009 and 2012/2013 DENV outbreaks, as well as control samples, were tested for the presence of DENV RNA by TMA. None of the samples collected during local dengue outbreaks were positive for DENV RNA (zero estimate, with a one-sided 95% CI: 0–0.06%), despite a subset being collected from “higher-risk” areas (Table 2). All of the control samples were also negative for DENV RNA (zero estimate, with a one-sided 95% CI: 0–0.23%).

## 4. Discussion

Dengue is an emerging disease of global significance and a current concern for the international transfusion community given the increasing number of transfusion transmitted (TT) cases [10–13]. DENV viremia has been detected in blood donors from Honduras, Puerto Rico, and Brazil, highlighting the potential risk for TT-DENV [25]. Countries have different risk mitigation approaches for managing the risk of TT-DENV, which in part depend on the level of dengue endemicity, the “risk appetite” of local clinicians and the public, and the size of their healthcare budget. These and additional factors should be considered in risk-based decision-making for blood safety. Currently, in Australia, donations of fresh blood components are restricted from “at-risk” donors travelling from areas where DENV transmission occurs, both within Australia and overseas. In this study, we were unable to detect DENV viremia in blood donors residing in areas of Australia experiencing local DENV outbreaks. Given that the upper confidence interval from this study was 0.06% (1 in 1,667), the current precautionary strategy of restricting “at-risk” donors to donating plasma for fractionation only is reasonable to mitigate the risk of a viremic donation.

None of the donors tested had detectable DENV infection, as evidenced by the absence of detecting either DENV RNA or DENV antigen. These results are concordant with earlier studies during previous outbreaks [4]. We previously estimated the risk of collecting a viremic donation during the 2008/2009 DENV outbreak to be 1 in 7,147 (95% CI: 1 in 2,218 to 1 in 50,021) [22], and modelling based on notification data obtained during a DENV outbreak in 2004 estimated the overall transmission risk to be 1 in 19,759 (95% CI: 1 in 3,404 to 75,486) with a peak of 1 in 5,968 (95% CI: 1 in 1,028 to 22,800) [26]. While this previous data suggests a low likelihood of finding a viremic sample in our study the absence of detectable evidence of DENV infection in donors, despite the higher number of reported cases during these outbreaks [6, 8], provides reassurance that our existing risk modelling does not substantially underestimate the risk of TT-DENV. While future studies using a larger sample size would refine the risk estimate, the small donor population in areas of Australia with DENV transmission means that such studies would not be practicable.

In Australia TT-DENV risk is mitigated through donation restrictions for “at-risk” donors, which has the potential to impact on availability of blood components for clinical use. Therefore, a blood donation screening assay that is capable of detecting donations containing an infectious virus could be used as an alternative. DENV antibody assays (IgM/IgG) would not be suitable for such a purpose, as DENV IgM is typically not present within blood until 3–5 days after the presentation of clinical symptoms and DENV IgG is not present for 1–14 days [27]. Detection of DENV antigen could be suitable for detecting asymptomatic and early infections in blood donors, as high levels of the antigen NS1 have been shown to be detectable within 72 hours of disease onset [28] and the assay format can be applied for high-throughput use. In this study two DENV NS1 antigen detection assays were used to determine the efficacy of detecting early DENV infection. We found discordant results between the two assays used, which is consistent with other studies. The Platelia Dengue NS1 Ag kit has been shown to have a higher sensitivity compared to the PanBio Dengue Early ELISA [23, 29, 30]. Although NS1 has been suggested as a useful tool in early DENV screening, sensitivity and specificity remain a major concern. Blood donor samples collected during a DENV outbreak in 2010/2012 in Puerto Rico were initially tested for NS1 antigen and later tested for DENV RNA (with TMA) to assess the possibility of TT-DENV from blood donors [25]. This study found that only 20% of RNA positive donor samples were positive on the Platelia Dengue NS1 Ag Kit, resulting in 42 patients being transfused with DENV RNA positive components [25]. This highlights the limitations of using DENV NS1 detection as a basis for blood donation screening assays for detecting DENV [25]; however, it should be noted that DENV NS1 still has a place in diagnostics. Therefore, detection of DENV RNA appears to be the most suitable for blood donation screening for DENV; however, testing may become costly. With the absence of detectable DENV RNA in any of the samples tested in this study, our current strategy of restricting fresh components from “at-risk” donors but continuing to collect plasma for fractionation appears an effective method for reducing the risk of TT-DENV in Australia. The strategy is also cost-effective because of the increasing demand in Australia for plasma to manufacture plasma derived immunoglobulin products [22]. Such an approach may also be suitable in other nonendemic areas, particularly those that experience episodic outbreaks and where source plasma is collected.

The levels of DENV viremia in blood donors during DENV outbreaks in Puerto Rico, Brazil, and Honduras (all considered endemic for dengue) were 0.19% [12], 0.04% [4], and 0.3% [4], respectively. Interestingly, DENV viremia was lower, 0.07%, in Puerto Rico in blood donors in a nonoutbreak period but during the seasonally heightened peak of dengue activity [41]. In contrast, no DENV viremic samples were found in Australian blood donors during the 2003 outbreak, although this study involved a relatively small number of donations from outbreak-affected areas [4]. The rate of dengue viremia in blood donors differs between areas endemic for DENV and also those considered episodic or nonendemic. Therefore, blood operators around the world require different risk-based decision-making strategies for dengue, depending on a number of factors including but not limited to the degree of dengue endemicity (endemic, episodic, and nonendemic); the acceptance of TT risk among local clinicians, as well as the public; the perceived severity of DENV infection among clinicians as well as the public compared to other infections; the proportion of the donor population travelling abroad and subject to travel-related blood donation restrictions; and, perhaps most importantly, the size of the healthcare budget. There are several approaches for reducing the risk of TT-DENV utilised by various blood operators across the world (Table 3). For example, in Hong Kong, where there is a constant high risk of dengue introduction from nearby mainland China [33], TT-DENV is mitigated through deferral of donors for 6 months who have previously had a dengue infection and a 2-week deferral for history of fever; however, no travel-related deferrals are in place [31]. However, in countries with minimal risk of DENV infection, such as New Zealand, TT-DENV is minimised through deferral of donors for 4 weeks who have previously had a DENV infection. In the future, other approaches may be used, for example, PI, particularly in areas endemic for dengue. The Theraflex UV-Platelets System (MacoPharma) has been shown to inactivate DENV in spiked platelet units to the limit of detection of the assay used [19], as has the Theraflex-MB Plasma System (MacoPharma) for DENV spiked into plasma [20]. Similarly the Intercept Plasma Inactivation System (Cerus Corporation) demonstrated inactivation of DENV in spiked plasma to the limit of detection [21]. In contrast the Mirasol PRT System (TerumoBCT) only partially inactivated DENV in spiked platelet units [42]. PI has the potential to assist with managing the TT-DENV risk in areas endemic for dengue or with episodic transmission and also has the potential to replace travel-related donation restrictions [43]. However, current technologies are not available for all blood products and therefore limit their application.

## 5. Conclusion

In this study we did not find evidence of DENV infection in the blood donors tested [6, 8]. The upper confidence interval of our blood donor viremia estimate of 1 in 1,667 suggests that the risk of collecting a viremic donation may be significant and supports the current precautionary strategy of restricting “at-risk” donors to donating plasma for fractionation only. It is clear that there is no overarching approach for the management of TT-DENV that is suitable for all countries. Each area should therefore assess TT-DENV risk based on local epidemiology and perform region-specific cost and risk analyses, which should collectively be considered in any future risk-based decision-making. The latter should consider that, as well as directly addressing transfusion risk, routine surveillance for infectious diseases in the blood donor population, including DENV, also serves as an important disease surveillance tool.

## Figures and Tables

**Table 1:** Detection of DENV NS1 in donations from Australian blood donors collected during local DENV outbreaks.

Sample	Platelia Dengue NS1 (# positive, # tested)	PanBio Dengue Early ELISA (# positive, # tested)	Overall result
1	N (0, 3)	P (3, 3)	Negative
2	N (0, 3)	P (3, 3)	Negative
3	N (0, 3)	P (3, 3)	Negative
4	E (1, 3) N (2, 3)	P (3, 3)	Negative
5	N (0, 3)	P (3, 3)	Negative
6	N (0, 3)	P (3, 3)	Negative
7	N (0, 3)	P (3, 3)	Negative
8	N (0, 3)	P (3, 3)	Negative
9	N (0, 3)	P (3, 3)	Negative
10	N (0, 3)	E (1, 3) P (2, 3)	Negative
11	N (0, 3)	P (3, 3)	Negative
12	N (0, 3)	P (3, 3)	Negative
13	N (0, 3)	P (3, 3)	Negative
14	N (0, 3)	P (3, 3)	Negative
15	N (0, 3)	P (3, 3)	Negative
16	N (0, 3)	P (3, 3)	Negative
17	N (0, 3)	E (1, 3) P (2, 3)	Negative
18	N (0, 3)	E (1, 3) P (2, 3)	Negative
19	N (0, 3)	P (3, 3)	Negative
20	N (0, 3)	P (3, 3)	Negative
21	N (0, 3)	P (3, 3)	Negative
22	N (0, 3)	P (3, 3)	Negative
23	N (0, 3)	E (1, 3) P (2, 3)	Negative
24	N (0, 3)	P (3, 3)	Negative
25	N (0, 3)	P (3, 3)	Negative
26	N (0, 3)	P (3, 3)	Negative
27	N (0, 3)	P (3, 3)	Negative
28	N (0, 3)	P (3, 3)	Negative
29	N (0, 3)	P (3, 3)	Negative
30	N (0, 3)	P (3, 3)	Negative
31	N (0, 3)	P (3, 3)	Negative
32	N (0, 3)	E (1, 3) P (2, 3)	Negative

N = negative, P = positive, and E = equivocal.

**Table 2:** Detection of DENV RNA, by TMA, in donations from Australian blood donors.

Samples	# tested	DENV RNA positive		
		#	%	95% CI
Dengue outbreak (2008/2009 and 2012/2013)	6,182	0	—	0–0.06
2008/2009 DENV epidemic	664	0	—	0–0.55
2012/2013 DENV outbreak	5,518	0	—	0–0.07
Control region	1,601	0	—	0–0.23

**Table 3:** Dengue endemicity and approaches used by blood operators for managing TT-DENV risk. Adapted from Teo et al., 2009 [31].

Country	Endemicity	Management approach
Australia	Nonendemic/episodic outbreaks in Queensland [6, 8]	(i) 4-week deferral for history of dengue infection [16] (ii) 4-week deferral for persons returning from dengue affected areas [16]
Canada	Nonendemic	3-week travel-related deferral for travel outside of Canada, continental USA, or Europe [32]
Hong Kong	Nonendemic [33]	(i) 6-month deferral for history of dengue infection [31] (ii) 2-week deferral for history of fever [31] (iii) No travel-related deferral for dengue [31]
Netherlands	Nonendemic	(i) 2-week deferral for history of dengue infection [34] (ii) 2-week deferral for history of fever [34] (iii) 4-week travel-related deferral for donors returning from dengue risk areas [34]
New Zealand	Nonendemic	(i) 4-week deferral for history of dengue infection [35] (ii) No travel-related deferral for dengue [35]
Puerto Rico	Endemic [36]	Pathogen inactivation (Intercept) recently implemented for use on plasma and platelet products [37]
Singapore	Endemic [38]	(i) 6-month deferral for history of dengue infection [31] (ii) 3-week deferral for history of fever [31] (iii) No travel-related deferral for dengue [31]
Sri Lanka	Endemic [39]	(i) No specific deferral for history of dengue infection [31] (ii) 2-week deferral for history of fever [31] (iii) No travel-related deferral for dengue [31]
United Kingdom	Nonendemic	(i) 2-week deferral for history of dengue infection [31] (ii) No travel-related deferral for dengue [31]
United States of America	Nonendemic/episodic outbreaks in some states [8, 40]	(i) 4-week deferral for history of dengue infection [31] (ii) No travel-related deferral for dengue [31]
